# Methanotrophic Communities and Cultivation of Methanotrophs from Rice Paddy Fields Fertilized with Pig-livestock Biogas Digestive Effluent and Synthetic Fertilizer in the Vietnamese Mekong Delta

**DOI:** 10.1264/jsme2.ME24021

**Published:** 2024-10-02

**Authors:** Huynh Van Thao, Mitsunori Tarao, Hideshige Takada, Tomoyasu Nishizawa, Tran Sy Nam, Nguyen Van Cong, Do Thi Xuan

**Affiliations:** 1 United Graduate School of Agricultural Science, Tokyo University of Agriculture and Technology, Tokyo 183–8509, Japan; 2 Department of Environmental Sciences, College of Environment and Natural Resources, Can Tho University, 3/2 street, Can Tho city 900000, Viet Nam; 3 Ibaraki University, College of Agriculture, 3–21–1 Chuou, Ami-machi, Ibaraki 300–0393, Japan; 4 Department of Microbial Technology, Institute of Food and Biotechnology, Can Tho University, 3/2 street, Can Tho city 900000, Viet Nam

**Keywords:** biogas digestive effluent, rice cultivation, methane emission, methanotroph community, methanotrophs, *pmo*A gene, Vietnamese Mekong Delta

## Abstract

Biogas digestive effluent (BDE) has been applied to rice fields in the Vietnamese Mekong Delta (VMD). However, limited information is available on the community composition and isolation of methanotrophs in these fields. Therefore, the present study aimed *(i)* to clarify the responses of the methanotrophic community in paddy fields fertilized with BDE or synthetic fertilizer (SF) and *(ii)* to isolate methanotrophs from these fields. Methanotrophic communities were detected in rhizospheric soil at the rice ripening stage throughout 2 cropping seasons, winter-spring (dry) and summer-autumn (wet). Methanotrophs were isolated from dry-season soil samples. Although the continued application of BDE markedly reduced net methane oxidation potential and the copy number of *pmo*A genes, a dissimilarity ordination ana­lysis revealed no significant difference in the methanotrophic community between BDE and SF fields (*P*=0.167). Eleven methanotrophic genera were identified in the methanotrophic community, and *Methylosinus* and *Methylomicrobium* were the most abundant, accounting for 32.3–36.7 and 45.7–47.3%, respectively. Type-I methanotrophs (69.4–73.7%) were more abundant than type-II methanotrophs (26.3–30.6%). Six methanotrophic strains belonging to 3 genera were successfully isolated, which included type I (*Methylococcus* sp. strain BE1 and *Methylococcus* sp. strain SF3) and type II (*Methylocystis* sp. strain BE2, *Methylosinus* sp. strain SF1, *Methylosinus* sp. strain SF2, and *Methylosinus* sp. strain SF4). This is the first study to examine the methanotrophic community structure in and isolate several methanotrophic strains from BDE-fertilized fields in VMD.

Biogas digestive effluent (BDE) is a byproduct of the methane fermentation process of livestock waste or biodegradable resources (Tang *et al.*, 2021). The process remains a vital nutrient constraint and increases pH (Bachmann *et al.*, 2014). BDE generally contains adequate essential nutrients and minerals that are suitable for plant growth (Fujiwara, 2012; Möller and Müller, 2012; Kumar *et al.*, 2015; Oritate *et al.*, 2016; Xu *et al.*, 2021; Chang *et al.*, 2022). It is recommended for use as an organic fertilizer alone or in combination with inorganic fertilizers (Zhang *et al.*, 2021). Recent studies demonstrated the feasibility of utilizing BDE for rice paddy fields (Win *et al.*, 2015; Oritate *et al.*, 2016; Minamikawa *et al.*, 2019). In this approach, BDE may not only reduce dependence on chemical fertilizers, but also recirculate unutilized nutrient resources for sustainable agricultural production.

Consistent with the preponderance of available nutrients, BDE anchors a labile carbon source (Minamikawa *et al.*, 2021; Tang *et al.*, 2021) and various complex organic compounds (*i.e.*, volatile fatty acids) (Cao *et al.*, 2016; Wang *et al.*, 2022a). Therefore, the application of BDE may drive the modification of soil properties. Previous studies revealed that the application of BDE increased the soil organic carbon (SOC) content (Win *et al.*, 2010; Xu *et al.*, 2019; Głowacka *et al.*, 2020; Tang *et al.*, 2021), available P and exchangeable Mg (Minamikawa *et al.*, 2021), pH, sorption properties (cation exchange capacity [CEC] and exchangeable cations [EAC]), K and Zn (Głowacka *et al.*, 2020), antibiotic accumulation and resistance genes (Lu *et al.*, 2021), and microbial diversity (Xu *et al.*, 2019; Zhang *et al.*, 2021) and suppressed soilborne diseases (Wang *et al.*, 2022a). However, other studies showed that the application of digestates did not affect the SOC content, whereas the enzyme activity of soil microorganisms was reduced (Bachmann *et al.*, 2014). Similarly, Minamikawa *et al.* (2021) reported no significant differences in pH, organic C, total N, exchangeable K, exchangeable Ca, or CEC. This discrepancy may be partly attributed to differences in BDE characteristics, the short/long-term application of BDE, and the practicing technology. Therefore, further studies are needed on the responses of rice paddy soil to the application of BDE.

The Vietnamese Mekong Delta (VMD) located in the southwest of Vietnam accounts for 53.5% of national rice productivity (Statistical Yearbook of Vietnam, 2022). The application of BDE to rice fields has been performed in various localities in VMD. The primary benefit of applying BDE is reducing dependence on chemical fertilizers for rice without yield loss (Minamikawa *et al.*, 2019; Huynh *et al.*, 2022). However, higher CH_4_ emissions in BDE fields versus SF fields (~19%) were reported in VMD (Minamikawa *et al.*, 2021). Reductions in CH_4_ emissions were agreed upon at the 2023 United Nations Climate Change Conference of the Parties of the UNFCCC (COP28), and the Vietnamese government is promoting the Action Plan for Methane Emissions Reduction by 2030. Therefore, solving the problem of CH_4_ emissions is critical to popularizing the application of BDE to rice cultivation in VMD.

Higher CH_4_ emissions in BDE fields is closely associated with the enrichment of labile organic carbon in BDE, which is a preferred substrate of methanogens (methane-producing archaea). Alternatively, the application of BDE stimulates the growth of methanogens. In contrast, the growth of methanotrophic populations (methane-oxidizing bacteria) may be promoted by increases in soil surface-generated CH_4_, which is a source of carbon and energy for methanotrophs, or reduced by oxygen deficiency. This argument has remained unclear in fields fertilized with BDE. Therefore, a more detailed understanding of the responses of the methanotrophic community in CH_4_ emission-related BDE fields is important for suggesting practical approaches to alleviate CH_4_ emissions in the future.

Methanotrophic microbes belong to the phyla *Proteobacteria* and *Verrucomicrobia* and Candidatus phylum NC-10 (Ettwig *et al.*, 2010; Dedysh and Knief, 2018; Rahalkar *et al.*, 2021). Methanotrophs are ubiquitous in nature and may be isolated from abundant environments (Cui *et al.*, 2020; Davamani *et al.*, 2020). The latest modified nitrate mineral salts (NMS) medium with a higher nitrate concentration, as recommended by Pandit *et al.* (2016), was recently used to successfully isolate seven genera from India’s rice paddy fields (Rahalkar *et al.*, 2021), and, thus, has potential in the identification of methanotrophs from the diverse features of rice paddy fields in tropical regions. Moreover, numerous methanotrophic strains remain uncultured, which has recently attracted considerable research interest (Dedysh and Knief, 2018; Guerrero-Cruz *et al.*, 2021). A more detailed understanding of the methanotrophic community structure and indigenous methanotrophic isolation may be helpful for future strategies to reduce the emission of greenhouse gases (GHG) from rice paddy fields (Pandit *et al.*, 2016; Davamani *et al.*, 2020). Therefore, the isolation of methanotrophs is more important for future strategies to reduce indigenous methanotroph-based CH_4_ emissions in agriculture production, particularly in rice fields fertilized with BDE from which CH_4_ emissions are higher.

Therefore, we herein aimed *(i)* to examine the methanotrophic community structure of rice fields fertilized with BDE or SF and *(ii)* to isolate methanotrophic strains in these fields in VMD. To achieve these goals, rhizospheric rice soil samples in fields fertilized with BDE or SF were collected to examine soil physiochemical properties, the copy number of *pmo*A genes (gene abundance in methanotrophs), and the methanotrophic community composition and to isolate methanotrophs from these rice fields.

## Materials and Methods

### Study site

Rice paddies in the Vinh Thanh district, Can Tho city (10°15′2.89″N; 105°18′32.27″E) were selected as a representative area of intensive rice production in VMD (Fig. S1). The study site was a low-lying area in a tropical latitude with a monsoon climate zone. The climate region is divided into two specific seasons. The dry season varies from November to April. The wet season is from May to October, accounting for 80% of annual precipitation (Tran *et al.*, 2019). The monthly average temperature, precipitation, and sunshine in the dry season (2020–2022) were 27.5°C, 70.9‍ ‍mm, and 199.7 h, respectively, while those in the wet season were 26.2°C, 219.9‍ ‍mm, and 139.7 h, respectively, (Dang and Hung, 2023).

### Soil sampling sites

Rhizospheric soil samples were collected from rice paddy fields fertilized with BDE or SF. These rice fields were intensive rice farming with 3 crops per year. In fields fertilized with BDE, the farmer diluted BDE with surface water from a nearby river prior to the irrigation of rice fields through a BDE storage pond. The characteristics of diluted BDE applied to the field are shown in Table S1. These fields were sown with the rice variety OM18, a typical rice cultivar used in VMD, at a sowing rate of 192.3‍ ‍kg ha^–1^. The maturity of this cultivar varies between 95–100 days. The water level was managed based on alternate wetting and drying (AWD) by the farmer (–10 to 10‍ ‍cm), which is a typical water management method as described by Uno *et al.* (2021). In SF fields, the total amount of N-P_2_O_5_-K_2_O fertilizers based on urea, NPK, and DAP (diammonium phosphate) was applied as follows: 138.5‍ ‍kg N ha^–1^, 88.25‍ ‍kg P_2_O_5_ ha^–1^, and 80‍ ‍kg K_2_O ha^–1^. The fertilizer was applied to rice fields 4 times: 8, 18, 28, and 38 days after sowing (DAS). In BDE fields, diluted BDE was applied every 10 days from 8 to 68 DAS (7 times per crop). These samples were taken in the ripening stage of the Winter-Spring season (Dry) (Dec 2021–Mar 2022) and Summer-Autumn season (Wet) (Apr–Jul 2022). The water level of the fields varied between –5 to 0‍ ‍cm from the soil surface. Five soil samples in each field were collected from rhizospheric areas by uprooting rice plants with the entire root systems and soil attached. The fresh weight of each sample was taken at approximately 0.5‍ ‍kg. Collected samples were immediately placed in airtight sterile polyethylene (PE) sampling bags and stored in a Styrofoam box at 4°C. After being transported to the laboratory, samples were quickly shaken off to remove soil from the root in a laminar flow hood. A similar portion of soil samples collected in the same field was mixed well to achieve a pooled sample correspondingly for isolating methanotrophs. Twenty samples were used to analyze soil physicochemical properties, and DNA was extracted for quantitative PCR (qPCR) and amplicon sequencing (5 samples per field×2 fields×2 seasons). The soil texture of BDE fields (*n*=*5*) consisted of clay 65±5.0%, silt 15±5.7%, and sand 20±3.7%, while that of SF fields was clay 64±4.1%, silt 16±4.1%, and sand, 20±3.10%. The soil of BDE and SF fields was classified as Gleysol based on World Reference Base (WRB) systems (IUSS Working Group WRB, 2015).

### Preparation of media

All methanotrophic bacteria were cultivated in NMS medium modified by Pandit *et al.* (2016) and Rahalkar *et al.* (2021) from Whittenbury *et al.* (1970). Medium was prepared as follows: MgSO_4_ 7H_2_O, 1‍ ‍g‍ ‍L^–1^; CaCl_2_, 0.2‍ ‍g‍ ‍L^–1^; KNO_3_, 0.25‍ ‍g‍ ‍L^–1^; SL10 solution, 1‍ ‍mL L^–1^; Fe_3_NH_4_ citrate solution, 1‍ ‍mL L^–1^; HEPES buffer (2 mol L^–1^, pH 7), 1‍ ‍mL L^–1^; post-autoclave additions included phosphate buffer pH 6.8, 2‍ ‍mL L^–1^ and vitamin solution (1×), 10‍ ‍mL L^–1^ (Rahalkar *et al.*, 2021). The solidifying agent was 2% agarose in medium to streak methanotrophs.

### Enrichment and isolation

Enrichment was conducted in a series of sterile 50-mL glass bottles. Ten serial dilutions (10^–1^–10^–10^) were implemented by adding 1.5‍ ‍g of each pooled soil sample to 13.5‍ ‍mL of sterilized NMS medium. The bottle was then closed airtight with a butyl rubber septum assembled with an aluminum crimp seal. Each bottle’s headspace air was flushed with nitrogen gas (99.999% N_2_, [v/v]) for approximately 3‍ ‍min (0.1 kPa). The enrichment experiment was performed under 20% CH_4_, 25% O_2_, and 55% N_2_. All bottles were then incubated at 25°C for two months in a dark place under static conditions (Rahalkar *et al.*, 2021). Methane concentrations were checked every 10 days, and the gas phase was replaced simultaneously to avoid oxygen depletion. Positive enrichment bottles were simultaneously identified by a decline in the content of CH_4_ and visual turbidity.

Fifty microliters of positive cultures was streaked on a 2% agarose plate of NMS and then incubated in air-tight chambers under a 20% CH_4_ and 80% air atmosphere. Isolated colonies that grew on the agarose plate were picked up using sterilized toothpicks/inoculation loops and transferred to 2-mL NMS in microtiter plates. These microtiter plates were then continuously incubated under conditions similar to those described above. After incubating for ~4‍ ‍weeks, positive wells were continuously transferred to 50-mL glass bottles to assess methane consumption. Positive bottles were constantly re-streaked on agarose plates for ~4‍ ‍weeks to obtain single colonies. These cycles were repeated several times until pure methanotrophic strains were obtained. The purity check was performed on several heterotrophic media (Pearlcore E-MC35; Bacto^TM^ Tryptic Soy Broth) and nutrient agar (Pearlcore E-MC63; Difco^TM^ R2A agar) (Rahalkar *et al.*, 2021). No growth on media indicated purity. The cells of methanotrophs were also observed using a transmission electron microscopy (TEM) (JEM-1400 Flash; JEOL) by negative staining. The bacterial culture medium was dropped onto a formvar-supported copper 200 mesh, which was held with self-locking fine forceps. The droplet on the grid was immediately dried using filter paper. EM stainer (Nisshin EM, #311) diluted ten times with ultrapure water was then dropped onto the grid. The droplet on the grid was immediately dried using filter paper. After drying, the drop was observed by TEM at 100 kV. The cells of methanotrophs were captured using a differential interference contrast microscope under 1000× magnification.

### Measurements

#### Methane oxidation potential (MOP)

The net soil MOP was measured by adding 5‍ ‍g of fresh soil to a 20-mL glass bottle that contained 10-mL sterile distilled water in triplicate (Yang *et al.*, 2022). The CH_4_ concentration was initially set by ~10% of the headspace. Bottles were placed on a shaker set at 120‍ ‍rpm in a dark place at 25°C. CH_4_ concentrations were measured after 0, 8, 16, and 36‍ ‍h (Fig. S2).

#### Soil physiochemical characteristics

Soil texture was exami­ned using the Pipette Robinson method (Carter and Gregorich, 2008). pH and electrical conductivity (EC) were extracted at a 1:5 ratio (1‍ ‍g of soil:5‍ ‍mL of distilled water) and detected using a glass pH electrode (9618S-10D; HORIBA Ltd.) and EC meter (ES-14; HORIBA Ltd.). Total organic carbon (TOC), total carbon (TC), and total nitrogen (TN) were detected using CN Corder (MT-700; Yanaco Technical Science), of which TOC was pretreated with concentrated HCl (37%) to eliminate all carbonates, followed by a 10-h dry step. Potential nitrogen mineralization (PNM) was measured by incubating soil anaerobically at 40°C for 7 days. NH_4_^+^ and NO_3_^–^ were then detected (Keeney and Bremner, 1966), extracted using 2 mol L^–1^ KCl, and assessed by the Indophenol blue method and Vanadium (III) reduction method, respectively (APHA, 1998).

Methane concentrations were measured with gas chromatography (GC-8A; Shimadzu) equipped with a flame ionization detector using the packed column (Porapak N, 80/100 mesh, 0.5 m). Injector, column, and detector temperature conditions were 180, 80, and 180°C, respectively. He gas was used as the carrier gas. Gas was taken directly from bottles using a 0.25-mL Pressure-Lok^®^ precision analytical syringe (VICI Precision Sampling).

#### DNA extraction from soil samples

DNA was extracted from 0.5‍ ‍g of fresh soil samples using the FastDNA^TM^ Spin Kit for soil (MP Biomedicals). DNA extraction procedures followed the manufacturer’s instruction manual. DNA concentrations were measured using a Qubit 4 Fluorimeter (Thermo Fisher Scientific) and stored at –80°C for further ana­lyses.

#### Amplicon sequencing ana­lysis of *pmoA* in soil samples

DNA templates were amplified using the universal *pmo*A primer (A189f and mb661r) (Holmes *et al.*, 1995; Costello and Lidstrom, 1999). The first PCR product was prepared in a 25-μL mixture as follows: 12.5‍ ‍mL Hot Start Taq 2× Master Mix (M0496L; New England Biolabs); 0.5‍ ‍μL each of 10‍ ‍μmol L^–1^ A189f and mb661r primers; 5‍ ‍μL of template DNA (1‍ ‍ng μL^–1^); and 6.5‍ ‍μL sterilized Milli-Q water. First PCR conditions were as follows: initial denaturation (94°C, 3‍ ‍mins); 30 cycles of denaturation (95°C, 30‍ ‍s), annealing (55°C, 30‍ ‍s), elongation (72°C, 30‍ ‍s); and final elongation (72°C, 10‍ ‍min) (Yang *et al.*, 2022). These PCR products were then sent to Bioengineering Lab.

First PCR products were purified with VAHTS DNA Clean Beads (Vazyme). Second PCR was performed for library quantification. The quality of the prepared library was confirmed using Analyzer and dsDNA 915 Reagent Kit (Agilent Technologies). Sequences were extracted using the fastx_barcode_splitter tool of FASTX-Toolkit (ver. 0.0.14). Sequences with a quality value less than 20 and a length <40 bases were removed using sickle (ver. 1.33). Reads were combined using the paired-end read merging script FLASH (ver. 1.2.11) with a minimum overlap of 10 bases. After removing chimera and noise sequences using the dada2 plugin of Qiime2 (ver. 2023.7), representative sequences were output with the amplicon sequence variants (ASV) table. Phylogeny was inferred from the representative sequence obtained against NCBI performing BLASTN (ver. 2.13.0). These procedures were conducted according to the standard protocols of Bioengineering Lab.

#### qPCR

The gene abundance of *pmo*A was analyzed by qPCR (Thermal cycler Dice Realtime System II systems, Takara Bio Inc.) using the specific primers A189f and mb661f. The qPCR product was prepared in a 25-μL mixture (12.5‍ ‍mL SYBR^®^ *Premix EX Taq 2X* (Takara Bio); 0.5‍ ‍μL each of 10‍ ‍μmol L^–1^ A189f and mb66r primers; 11.5‍ ‍μL of template DNA and sterilized Milli-Q water). qPCR conditions were as follows: initial denaturation at 95°C for 30‍ ‍s followed by 2-step PCR with 40 cycles of denaturation at 95°C for 5‍ ‍s and annealing at 60°C for 30‍ ‍s. Melting curve data were obtained by maintaining temperature at 95°C for 15‍ ‍s, lowering it to 60°C for 1‍ ‍min, then raising it to 95°C for 15 s. A serial standard curve was constructed from a DNA mixture of methanotrophs (*Methylosinus* sp. strain SF4 and *Methylocystis* sp. strain BE2) isolated in the present study. The PCR efficiency of *pmo*A was calculated to be 91.1%.

#### DNA extraction, amplification, and sequencing of isolated methanotrophs

DNA from isolated methanotrophs was extracted from pure cultures using ISOPLANT (Nippon Gene Co., Ltd.). DNA concentrations were measured using QUBIT procedures. Amplification was performed using primers comprising 27f–1492r for the 16S rRNA gene and A189f-mb661r for the *pmo*A gene (Rahalkar *et al.*, 2021) in a total volume of 50‍ ‍μL (25‍ ‍mL Hot Start Taq 2× Master Mix, 1‍ ‍μL each of 10‍ ‍μmol L^–1^ *(i)* 27f–1492r and *(ii)* A189f–mb661r primers, and 23‍ ‍μL of template DNA and sterilized MiliQ water). PCR conditions for the 16S rRNA gene were as follows: initial denaturation (94°C, 3‍ ‍min); 30 cycles of denaturation (95°C, 30‍ ‍s), annealing (46°C, 30‍ ‍s), and elongation (68°C, 90 s); and final elongation (68°C, 5‍ ‍min). PCR conditions for the *pmo*A gene were as follows: initial denaturation (94°C, 3‍ ‍min); 30 cycles of denaturation (95°C, 30‍ ‍s), annealing (55°C, 30 s), and elongation (72°C, 30‍ ‍s); and final elongation (72°C, 10‍ ‍min) (Yang *et al.*, 2022). PCR products were cleaned with Isospin PCR Product (Nippon Gene). Cleaned template DNAs were checked on gel electrophoresis with 1.5% agarose (Fig. S3). Amplified products were then outsourced for sequencing to Eurofins Genomics. The sequences acquired were aligned using Molecular Evolutionary Genetics Analysis (MEGA11) software and subjected to a BLAST ana­lysis with the 16S rRNA gene and *pmo*A gene using the NCBI database. Isolated methanotroph gene sequences (*pmoA* and 16S rRNA) were submitted to the NCBI database. The accession numbers (*pmo*A; 16S rRNA) of strains were as follows: *Methylococcus* sp. strain BE1 (PP182324; PP178148), *Methylocystis* sp. strain BE2 (PP239038; PP218659), *Methylosinus* sp. strain SF1 (PP239039; PP218660), *Methylosinus* sp. strain SF2 (PP239041; PP218670), *Methylococcus* sp. strain SF3 (PP239042; PP218671), and *Methylosinus* sp. strain SF4 (PP239040; PP218672).

#### Statistical ana­lysis

Statistical ana­lyses were performed using R version 4.3.2 (R Core Team [2023]—R Foundation for Statistical Computing, Vienna, Austria) with a confidence level of 95%. Soil physiochemical characteristics, MOP, and the copy number of *pmo*A genes among rice fields were tested using a principal component ana­lysis (PCA). The dataset was standardized using the “Scale” function to calculate the z-scores for each variable. A principal coordinate ana­lysis (PCoA) was performed to measure the dissimilarity matrices of the methanotrophic community among sampling sites using the Euclidean distance at genus levels. Dissimilarity ordinations between fields were exami­ned using a permutational multivariate ana­lysis of variance (PERMENOVA). A redundancy ana­lysis (RDA) was also performed to analyze the relationships between the community structures of methanotrophs and environmental variables. A step function was applied to eliminate the collinearity of explanatory variables and only select the best variables to simplify the model before performing RDA. Variances were standardized using Hellinger transformation before computing. PCoA and RDA computations were performed using the Vegan package (Version 2.6-4). Pearson’s correlation ana­lysis was used to investigate the relationships among environmental variables, MOP, *pmo*A, and methanotrophic community genus levels using the visualization of a correlation matrix (Corrplot, Version 0.92). Data were visualized using the ggplot2 package (version 3.4.4).

## Results

### Rhizospheric soil physiochemical properties, soil MOP, and gene abundance of methanotrophs (*pmoA*)

**Fig. 1.**
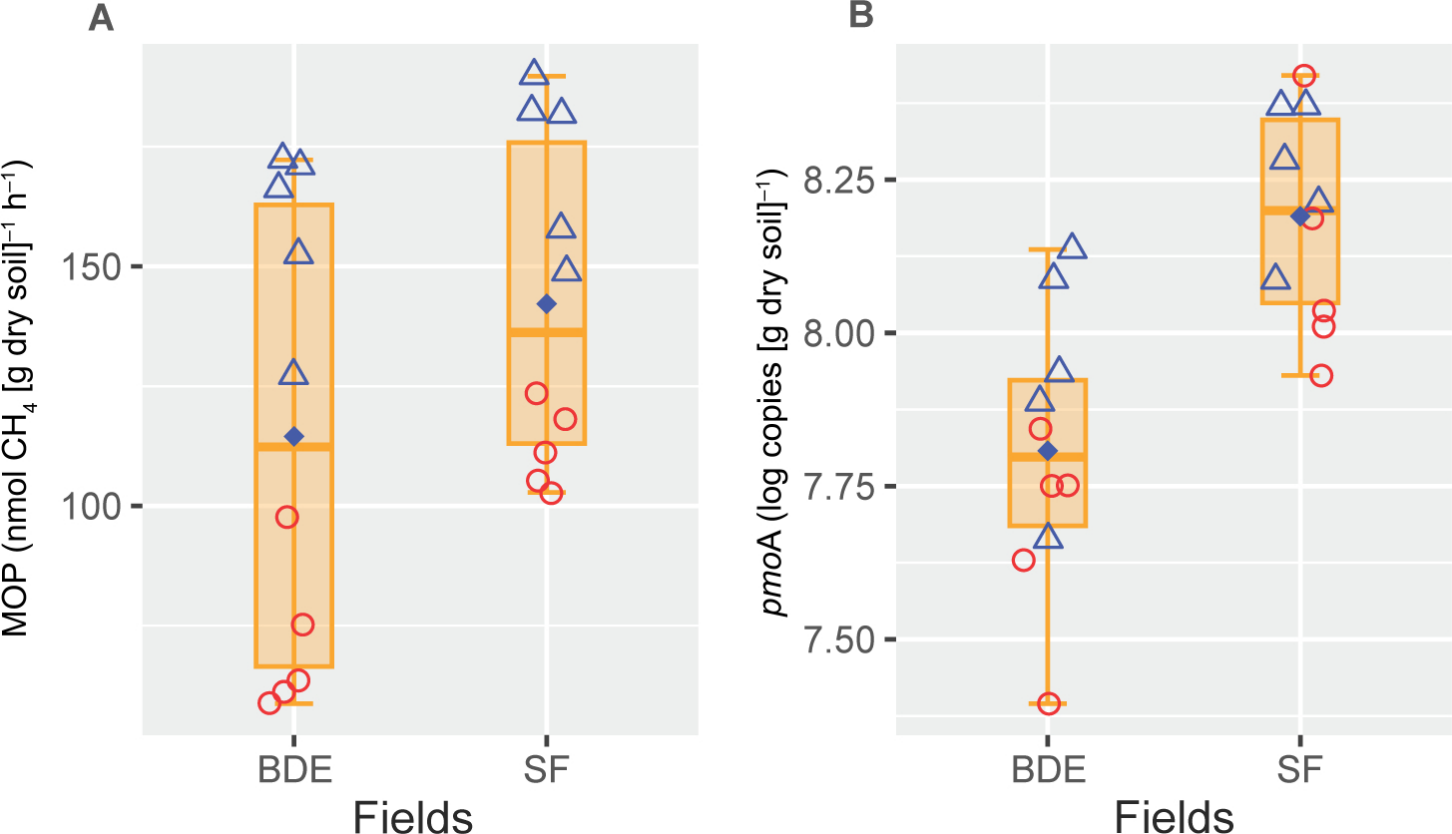
Methane oxidation potential (A) and copy numbers of *pmo*A genes (B) in BDE and SF fields. Symbols (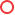 and 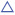) indicate the dry and wet season, respectively. Symbols in each boxplot indicate replications (*n*=10). Filled diamonds denote mean values in BDE and SF fields.

The physicochemical characteristics of soil rice rhizospheres fertilized with BDE and SF are shown in Table S2. Variations in rhizospheric soil properties slightly differed between the seasons and fields. Fig. 1. shows variations in soil MOP and the gene abundance of methanotrophs (*pmo*A). MOP values were in the range of 59–172 and 103–190‍ ‍nmol CH_4_ (g dry soil)^–1^ h^–1^ for BDE and SF fields, respectively (Fig. 1A). The mean MOP value in the BDE field (115‍ ‍nmol CH_4_ [g dry soil]^–1^ h^–1^) was lower than in the SF field (143‍ ‍nmol CH_4_ [g dry soil]^–1^ h^–1^). Furthermore, MOP values were higher in the dry season (165‍ ‍nmol CH_4_ [g dry soil]^–1^ h^–1^) than in the wet season (91.7‍ ‍nmol CH_4_ [g dry soil]^–1^ h^–1^). Similarly, *pmo*A gene abundance in BDE and SF fields varied between 2.4×10^7^ and 1.36×10^8^ and between 8.5×10^7^ and 2.6×10^8^ copies [g dry soil]^–1^, respectively (Fig. 1B). *pmo*A abundance was lower in the BDE field (7.2×10^7^ copies [g dry soil]^–1^) than in the SF field (1.7×10^8^ copies [g dry soil]^–1^). However, mean values slightly differed between the dry (1.4×10^8^ copies [g dry soil]^–1^) and wet (9.6×10^7^ copies [g dry soil]^–1^) seasons.

**Fig. 2.**
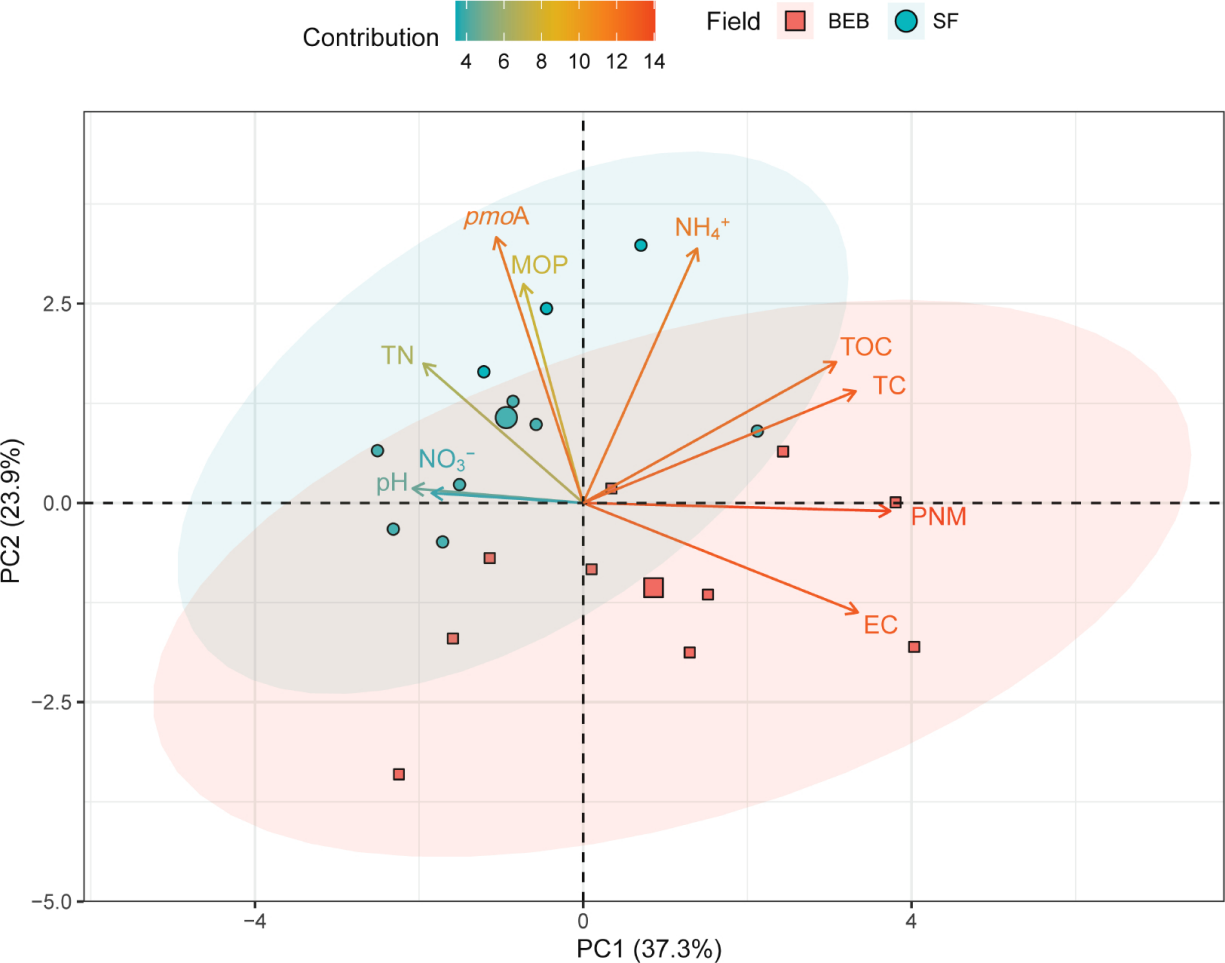
A principle component ana­lysis (PCA) of variables of soil physiochemical characteristics, methane oxidation potential, and the *pmo*A gene. Two PCs were extracted from dimensions that explained 61.2%. Ellipses indicate the dispersion of data points and principal components for each field. Larger ellipse shows greater variance. The change in arrow colors denotes the contribution of response variables in each dimension (PC).

The distribution of soil physicochemical characteristics was distinct in the present study (Fig. 2). Two PCs were extracted that accounted for 61.2% (37.3% PC1 and 23.9% PC2) of observed variables. The BDE field was characterized by high EC and PNM (PC1), and the SF field by higher NH_4_^+^ and *pmo*A (PC2).

### Methanotroph diversity and community composition of methanotrophs

**Fig. 3.**
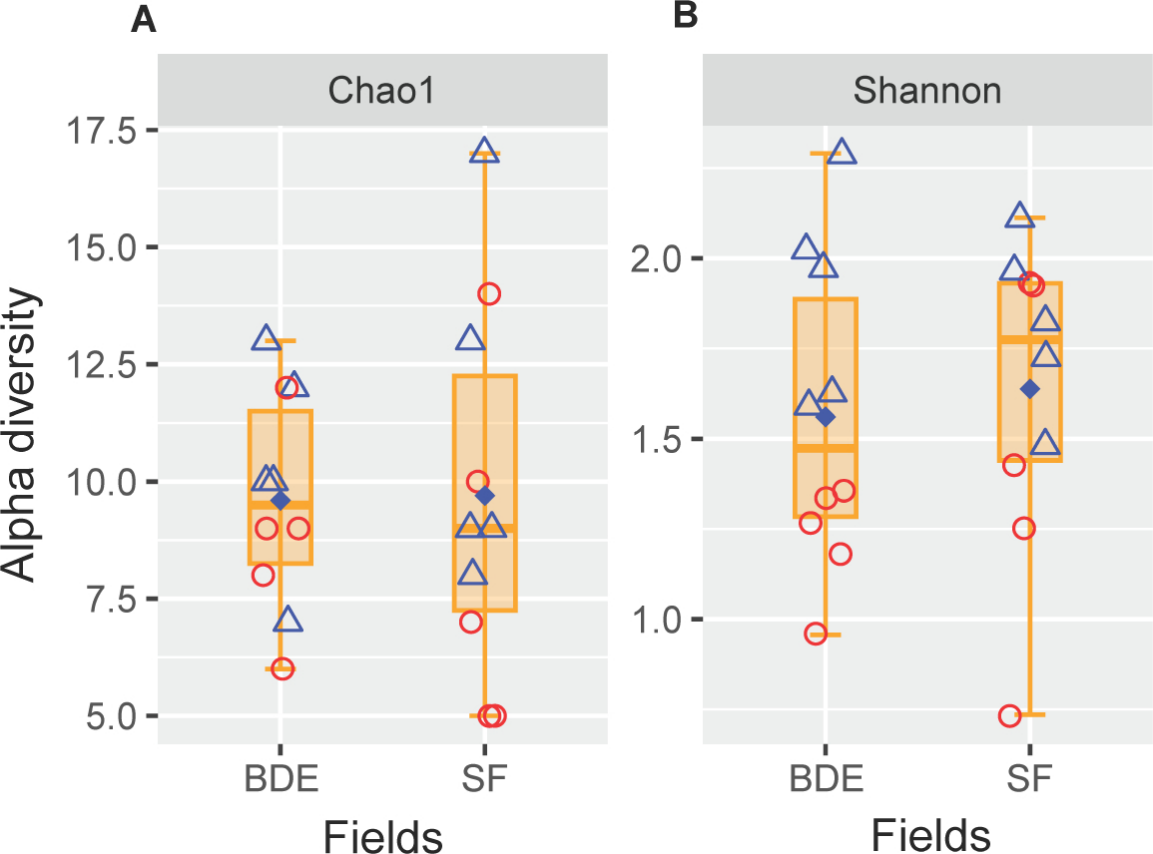
Alpha diversity at the genus level (Chao1 [A] and Shannon [B]) of the methanotroph community in BDE and SF fields. Symbols (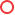 and 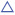) indicate the dry and wet season, respectively. Symbols in each box indicate replications (*n*=10). Filled diamonds denote mean values in BDE and SF fields.

The methanotrophic community was detected based on the amplicon sequencing ana­lysis of *pmo*A genes. A total of 1,122 high-quality *pmo*A gene ASVs were obtained for the BDE and SF fields, which resulted in 72 ASVs being identified for methanotrophs. The alpha diversity of the methanotrophic community is shown in Fig. 3. The richness of ASVs (Chao1) obtained in the BDE and SF fields were 6–13 and 5–17, respectively. The Shannon index was in the range of 0.96–2.29 and 0.74–2.11 for the BDE and SF fields, respectively. Correspondingly, the Chao1 index was in the range of 7–17 and 5–14 ASVs in the dry and wet seasons, respectively. The Shannon index was in the range of 0.63–0.88 and 0.74–1.93 for the dry and wet seasons, respectively.

**Fig. 4.**
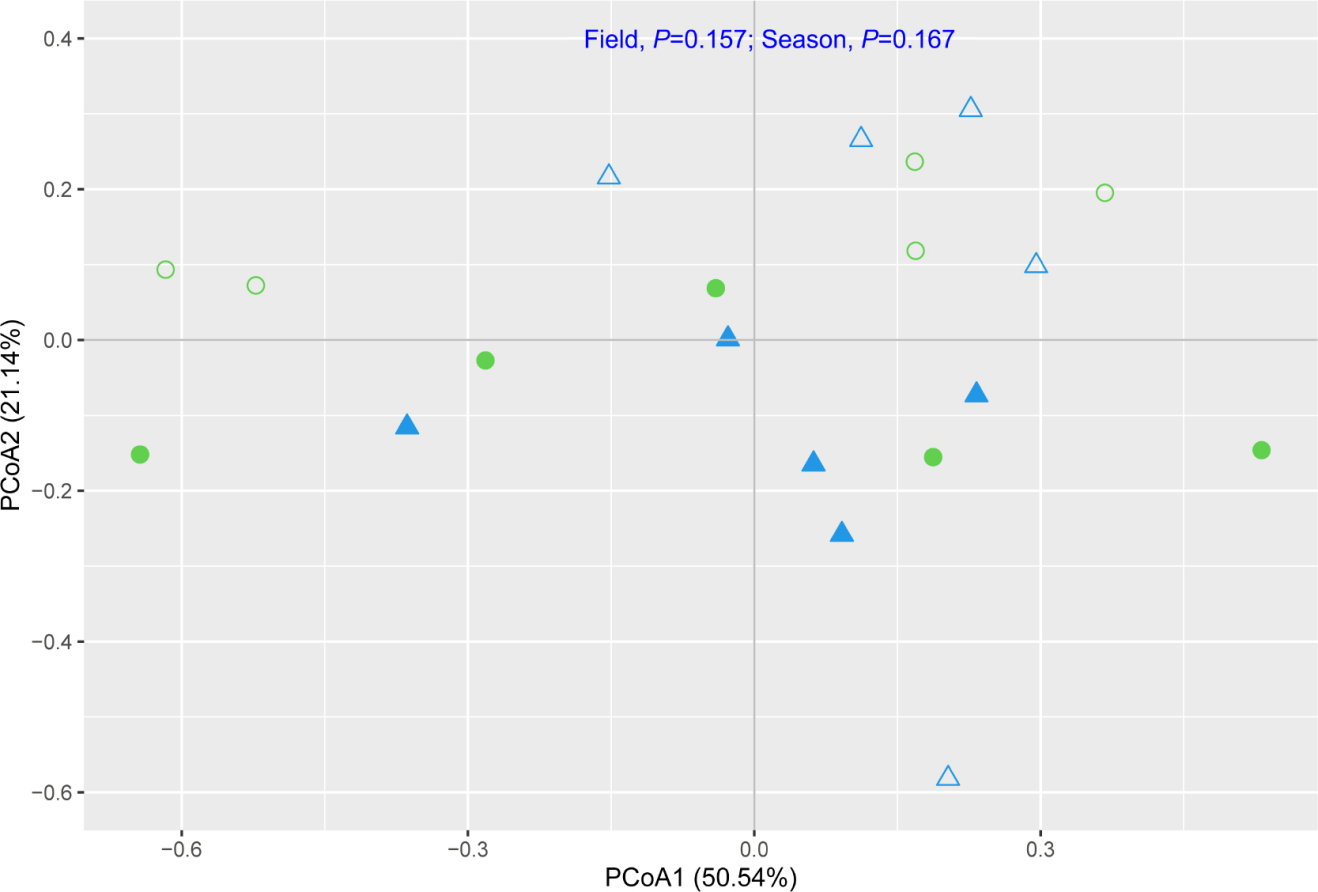
Principle coordination ana­lysis (PCoA) ordination diagram of the methanotrophic community at the genus level in BDE and SF fields. The methanotrophic community at the genus level in BDE (open) and SF (closed) fields in the dry (triangle) and wet (circle) seasons.

**Fig. 5.**
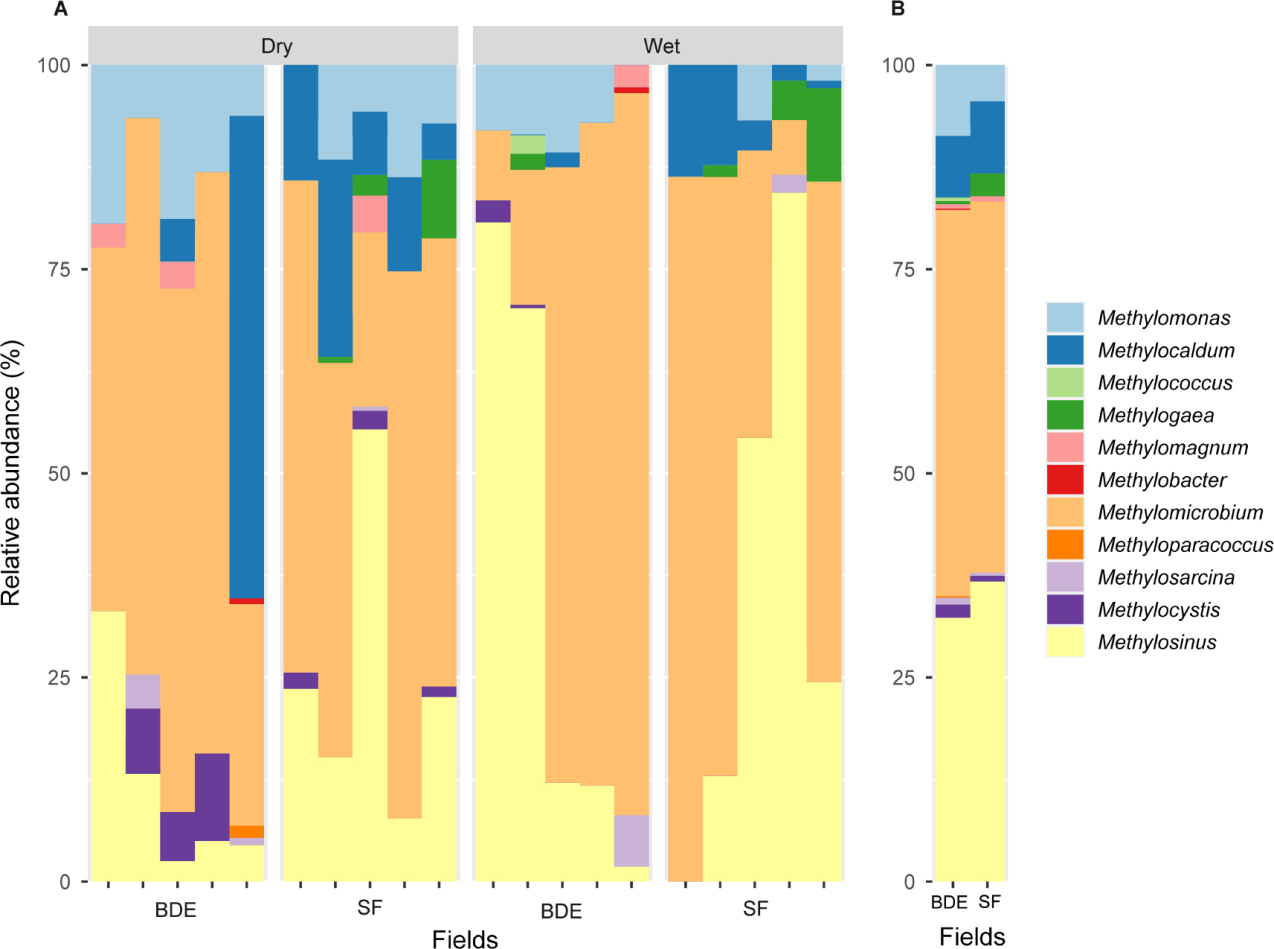
Methanotrophic community structure at the genus level in BDE and SF fields in dry and wet seasons. A and B indicate all samples and mean values, respectively.

Fig. 4 shows the PCoA of the methanotrophic community structure. PCoA results explained the structure with 71.68% (PCoA1, 50.54%; and PCoA2, 21.14%). These results revealed that the methanotrophic community did not significantly differ between the BDE and SF fields (*P*=0.157) or between the dry and wet seasons (*P*=0.167). Type-I methanotrophs comprising *Methylobacter*, *Methylocaldum*, *Methylococcus*, *Methylogaea*, *Methylomagnum*, *Methylomicrobium*, *Methylomonas*, *Methyloparacoccus*, and *Methylosarcina* and type-II methanotrophs encompassing *Methylosinus* and *Methylocystis* were present in the BDE and SF fields (Fig. 5A). Among genera, *Methylosinus* and *Methylomicrobium* were dominant in paddy soil. The mean percentages of *Methylosinus* and *Methylomicrobium* were in the range of 22.3–43.7 and 45.5–48.6% in the dry and wet seasons, respectively (Fig. S4). In comparisons of the BDE and SF fields, the average percentages of *Methylosinus* and *Methylomicrobium* was in the range of 45.7–47.3 and 32.3–36.7%, respectively (Fig. 5B). Type-I methanotrophs (69.4–73.7%) were more abundant than type-II methanotrophs (26.3–30.6%) (Fig. S5).

### Factors controlling the methanotrophic community in rice paddy fields

**Fig. 6.**
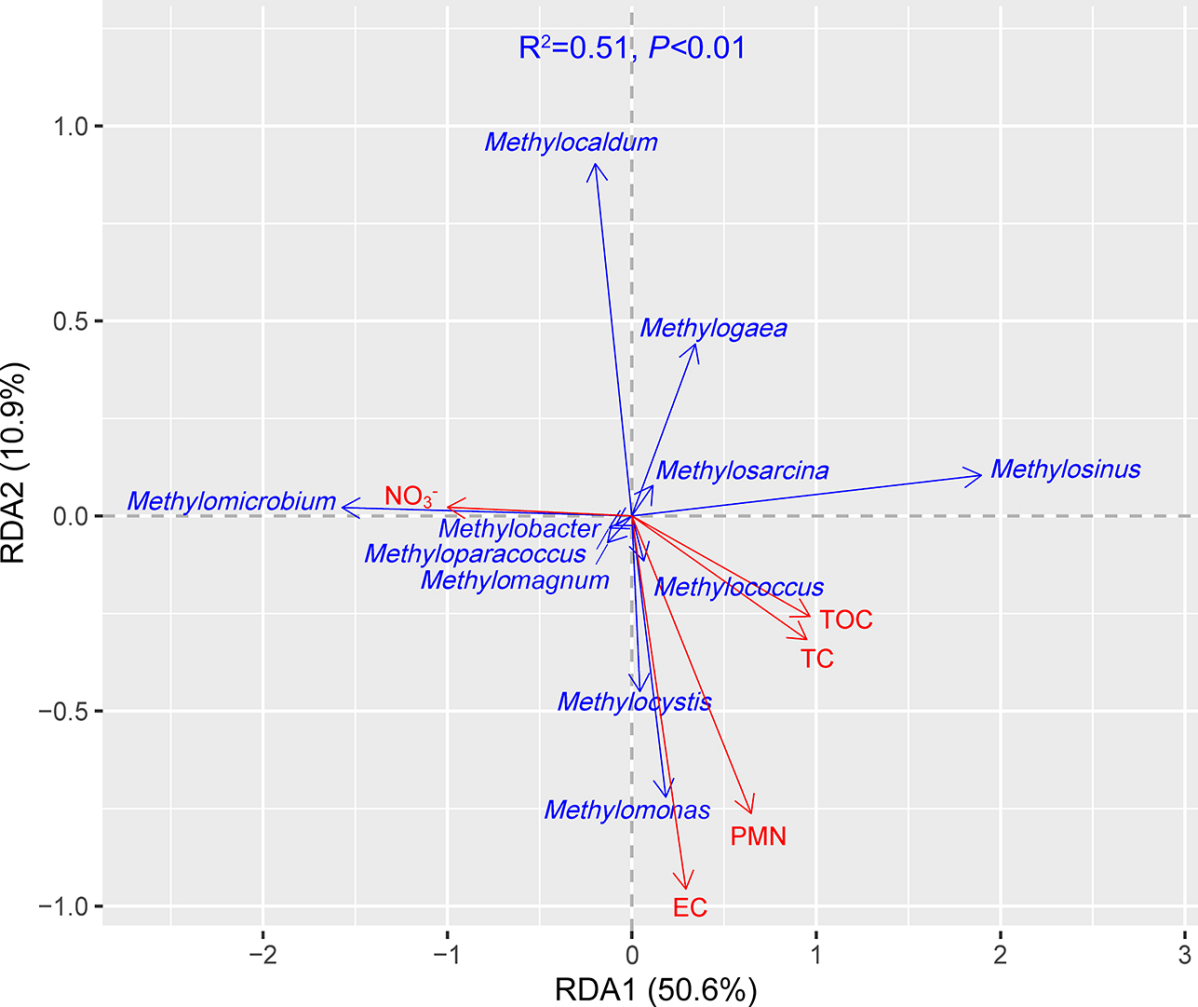
RDA ordination plot indicating the relationship between soil environmental variables and genus levels of the methanotrophic community. A step function was applied to the model in order to select the best variables, thereby simplifying the models.

RDA was performed to identify factors affecting the methanotroph community structure in soil (Fig. 6). The presented RDA model was significant (*P*<0.01). The percentages of explained variance of RDA1 and RDA2 were 50.6 and 10.9%, respectively, resulting in 61.5% of explained combined variance. RDA1 was significant for explaining the relationship between environmental and response variables (Table S3). EC (*P*<0.05), TOC (*P*<0.05), and NO_3_^–^ (*P*<0.01) had a significant effect on the community structure of methanotrophs at the genus level (Table S4).

**Fig. 7.**
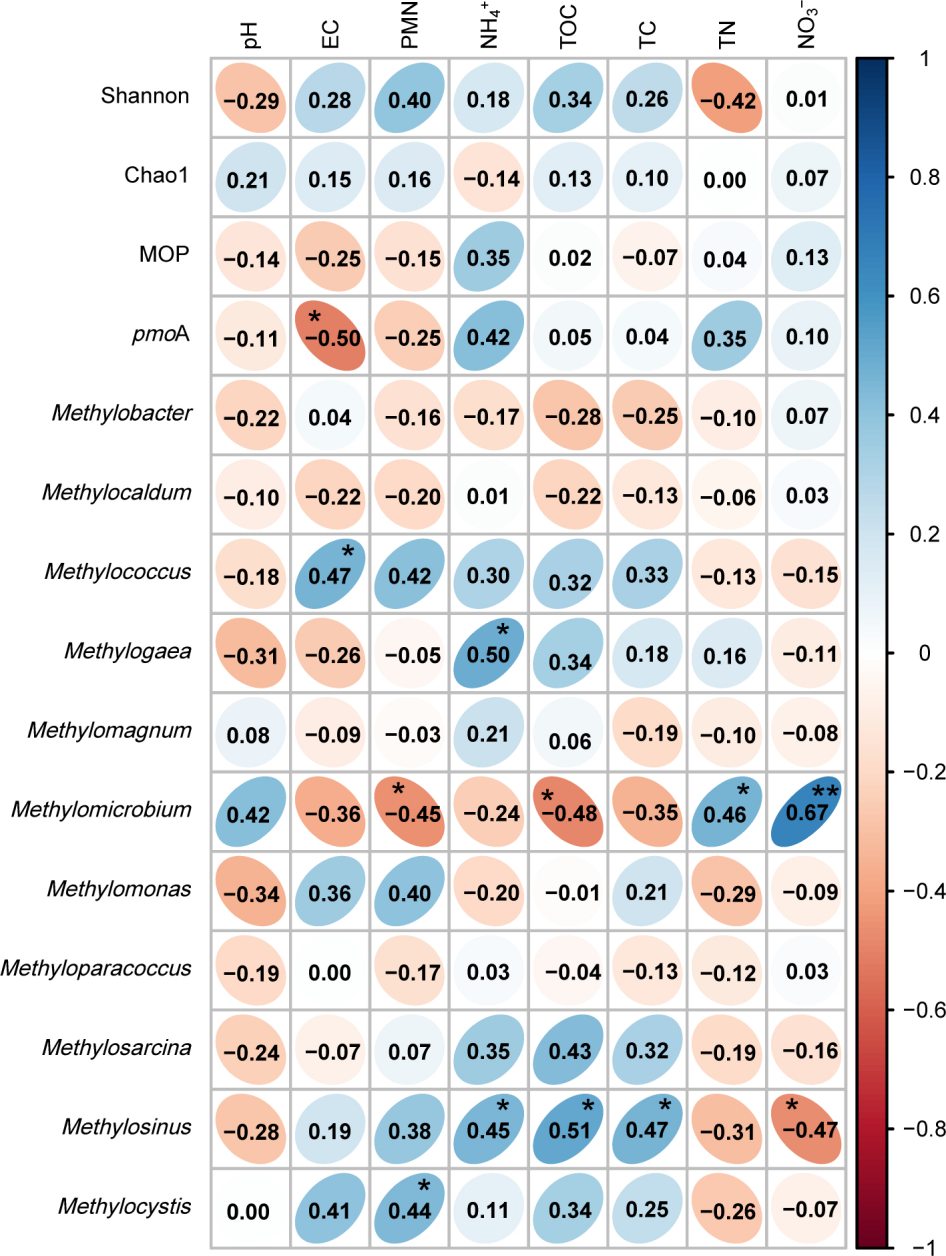
A heatmap of Pearson’s correlation coefficients between soil environmental variables and alpha diversities (Chao1 and Shannon indices), MOP, *pmo*A, and methanotrophic genus levels. Significance was tested using Pearson’s correlation coefficient. ***P<0.001, **P<0.01, *P<0.05, ^†^*P*<0.1.

Pearson’s correlation ana­lysis (Fig. 7) showed that *Methylomicrobium* negatively correlated with PNM (*P*<0.05) and TOC (*P*<0.05), but positively correlated with TN (*P*<0.05) and NO_3_^–^ (*P*<0.001). *Methylosinus* positively correlated with NH_4_^+^ (*P*<0.05), TOC (*P*<0.05), and TC (*P*<0.05), but negatively correlated with NO_3_^–^ (*P*<0.05). Similarly, *Methylogaea* positively correlated with NH_4_^+^ (*P*<0.05). *Methylocystis* and *Methylococcus* positively correlated with PNM and EC (*P*<0.05), respectively. *Methylosarcia*, *Methyloparacoccus*, *Methylomonas*, *Methylomagnum*, *Methylocaldum*, and *Methylobacter* did not correlate with any variables. Notably, *pmo*A gene abundance negatively correlated with EC (*P*<0.05), while MOP, the Chao1 index, and the Shannon index did not correlate with any variables.

### Isolation of methanotrophs from paddy soils

**Fig. 8.**
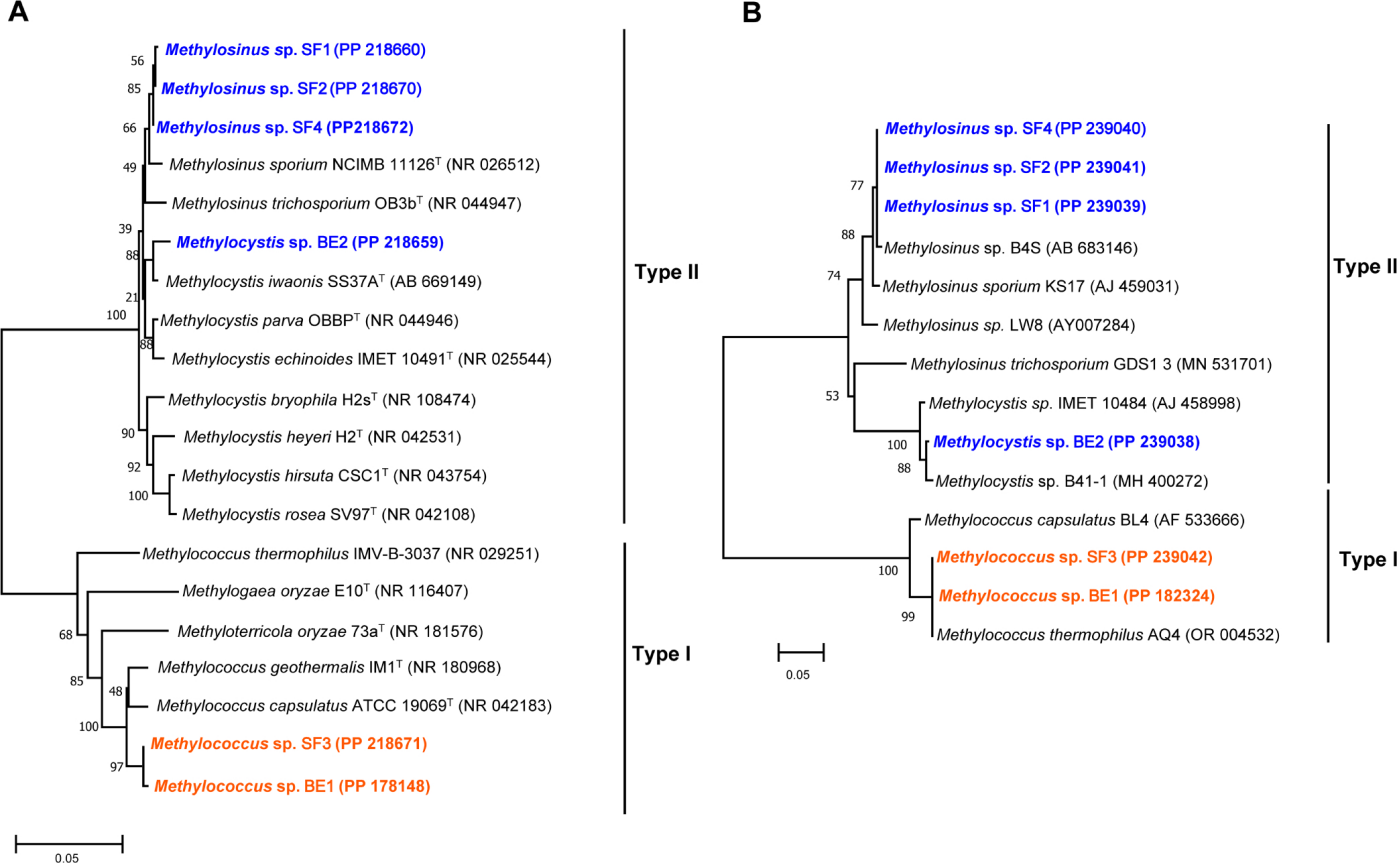
Neighbor-joining trees showing the polygenetic tree of isolated methanotrophs with their closest member based on 16S rRNA (A) and *pmo*A gene (B) sequences. Isolated strains are highlighted in orange (type-I methanotrophs) and blue (type-II methanotrophs). All ambiguous positions were removed for each sequence pair. Bootstrap (1,000 interactions) values are shown. The scale bar indicates 5% estimated sequence divergence.

The present study was conducted as a serial step for methanotroph cultivation. Methanotrophs generally grew under a dilution rate of 10^–1^–10^–8^, resulting in visual turbidity and the formation of pellicles accompanied by CH_4_ consumption. On average, 4–5 streaking steps resulted in a pure culture. The dilution rate to achieve axenic cultures varied between 10^–4^ and 10^–6^. Based on 16S rRNA (Fig. 8A) and *pmo*A sequences (Fig. 8B), 6 methanotrophic strains with 3 genera belonging to 3 clades of proteobacterial methanotrophs were identified. The strains of isolated methanotrophs included *Methylosinus*. sp. (3 strains), *Methylococcus* sp. (2 strains), and *Methylocystis* sp. (1 strain) (Table 1). *Methylocystis* sp. and *Methylosinus* sp., classified as type-II methanotrophs, were isolated from the BDE and SF fields, respectively, while *Methylococcus* sp., categorized as type-I methanotrophs, were isolated from the BDE and SF fields. The colors of methanotrophic colonies were white, cream and light yellow (Fig. S6). The cell morphology of isolated methanotrophs was ellipsoid (Fig. S7). Furthermore, the *pmo*A sequences of the isolated strains were similar to the *pmo*A gene amplicons identified in VMD rice fields (Fig. S8).

## Discussion

The present study collected rhizospheric soil from intensive paddy fields fertilized with BDE and SF, with BDE being applied for three years. Slight differences were observed in pH, TOC, TC, TN, NH_4_^+^, and NO_3_^–^ between the BDE and SF fields (Table S2). However, PCA results (PC1) revealed increases in EC and PNM with the application of BDE (Fig. 2). Diluted BDE utilized for rice paddy fields contained high levels of EC (22–26 mS cm^–1^), TOC (266–283‍ ‍mg L^–1^), and TN (234–278‍ ‍mg N L^–1^) (Table S1). The accumulation and mineralization of organic carbon and nitrogen following the application of a large amount of BDE markedly increased EC and PNM, which had not been reported in previous studies. The application of BDE did not affect the SOC content which is consistent with previous findings (Bachmann *et al.*, 2014; Minamikawa *et al.*, 2021), while the augmentation of EC and PNM was observed in the current study. Therefore, further studies need to consider the long-term application of BDE in order to change the content of SOC.

Previous studies reported that MOP and the copy number of *pmo*A genes markedly varied between rice paddy soils: ~20–396‍ ‍nmol CH_4_ [g dry soil]^–1^ h^–1^ and ~5.3×10^6^–1.5×10^8^ copies [g dry soil]^–1^, respectively (Shrestha *et al.*, 2010; Yang *et al.*, 2022). However, the effects of the application of BDE on MOP and *pmo*A in these fields remain unclear. The present results on MOP and *pmo*A were consistent with findings described above. Nevertheless, MOP and *pmo*A were significantly lower in the BDE field than in the SF field (PC2), indicating that the long-term application of BDE regulates the structure of the methanotrophic community. The application of BDE has been shown to suddenly increase the concentration of NH_4_^+^, thereby inhibiting methanotrophic metabolism due to the competitiveness of NH_4_^+^ versus CH_4_, resulting in lower MOP and methanotroph abundance (Yang *et al.*, 2022). Lower MOP and *pmo*A were not associated with higher CH_4_ emissions because of their contradictory impacts on CH_4_ production compared to CH_4_ oxidation (Shrestha *et al.*, 2010), albeit higher CH_4_ emissions were observed in BDE-fertilized fields (Huang *et al.*, 2014; Minamikawa *et al.*, 2021). On the other hand, the CH_4_ emission rate results from the interaction between active methanotrophs and methanogens in rice paddy ecosystems (Qian *et al.*, 2023). Consistent with this hypothesis, Win *et al.* (2016) reported that the application of biogas slurry to paddy fields increased CH_4_ emissions; however, the number of *pmo*A genes also increased over those in conventional rice cultivation. The transcription ratio of *pmo*A/*mcr*A (methanotrophs/methanogens) may more accurately predict CH_4_ emissions from fields. Universally, a higher ratio of *pmo*A/*mcr*A typically results in lower CH_4_ emissions in paddy fields (Lee *et al.*, 2014; Sakoda *et al.*, 2022). Although the present study did not examine the relationship between *pmo*A/*mcr*A and CH_4_ emissions, a positive correlation was observed between *pmo*A and MOP. Therefore, the relationships between MOP, *pmo*A/*mcr*A, and CH_4_ emissions need to be clarified in further investigations in VMD.

Recent studies reported that the application of biogas slurry changed the bacterial community structure in rice fields (Xu *et al.*, 2019; Wang *et al.*, 2022a; Wang *et al.*, 2022b), and described the methanotrophic community structure in rice ecosystems (Shrestha *et al.*, 2010; Phung *et al.*, 2021; Rahalkar *et al.*, 2021; Sakoda *et al.*, 2022; Yang *et al.*, 2022). However, the responses of methanotrophs under BDE fertilization has remained an obstacle. The present study revealed similarities in methanotrophic communities in BDE and SF fields (Fig. 4), indicating that the application of BDE did not significantly affect the community structure of methanotrophs. The results obtained also showed that type-I methanotrophs (9 genera) outcompeted type-II methanotrophs (2 genera) (Fig. S5), which was consistent with previous findings on rhizospheric soil (Wu *et al.*, 2009; Lüke *et al.*, 2010, 2014; Shrestha *et al.*, 2010), indicating that type-I methanotrophs were responsible for regulating CH_4_ emissions in these fields. Therefore, the application of BDE to rice fields may not significantly affect environmental conditions, particularly CH_4_ and O_2_, which support the thriving of the type-I methanotrophic community. Nevertheless, the present study only exami­ned the methanotrophic community structure of a single stage of rice ripening in these fields. Therefore, the dynamics of the methanotrophic community during rice growing need to be considered in VMD rice fields in further studies.

In rice paddy ecosystems, type-I methanotrophs change more rapidly under the revolution of fields than type-II methanotrophs (Sakoda *et al.*, 2022). Type-I methanotrophs prefer low CH_4_ concentrations and are sensitive to nitrogen fertilization, while higher CH_4_ concentration accelerate the growth of type-II methanotrophs (Takeda *et al.*, 2008; Knief, 2015; Yang *et al.*, 2022). Additionally, type-II methanotrophs (*i.e.*, *Methylosinus* sp. and *Methylocystis* sp.) may tolerate unfavorable environments, such as rice paddy ecosystems (Shrestha *et al.*, 2010). Among the genera identified in the methanotrophic community in the present study, *Methylomicrobium* (type I) and *Methylosinus* (type II) were the most abundant, indicating their positive activity in the methanotrophic community of rice fields as well as their potential role in controlling MOP. Among explanatory variables, EC, TOC, and NO_3_^–^ were primary factors that significantly impacted the methanotroph community (Fig. 6), with TOC and NO_3_^–^ being significant in the RDA1 model, which decisively affected *Methylosinus* and *Methylomicrobium*. EC was more closely associated with RDA2, which was not significant in the RDA model (Table S4). Pearson’s correlation ana­lysis revealed that *Methylosinus* positively correlated with NH_4_^+^, TOC, and TC and negatively correlated with TN and NO_3_^–^, while *Methylomicrobium* positively correlated with TN and NO_3_^–^ and negatively correlated with PNM and TOC (Fig. 7). These results indicate that the dynamics of the methanotrophic community interchanges according to the *Methylosinus* population with the enrichment of the carbon source coupled with NH_4_^+^ or the abundance of *Methylomicrobium* with increases in TN and NO_3_^–^. An increased carbon source is a preferred environment for methanogens, resulting in higher CH_4_ production (Qian *et al.*, 2023), which also benefits type-II methanotrophs (Shrestha *et al.*, 2010; Vishwakarma *et al.*, 2010; Sakoda *et al.*, 2022; Yang *et al.*, 2022), thereby explaining the present results.

The isolation of methanotrophs faces numerous challenges that have only been addressed by a few studies globally (Rahalkar *et al.*, 2021). In VMD, the isolation of methanotrophs from rice fields as well as the methanotrophic community structure have yet to attract the attention of researchers. This is the first study to isolate methanotrophs from rice paddy fields in VMD. Three methanotrophic genera were isolated from among the 11 methanotrophic genera identified in the methanotrophic community of VMD paddy fields. Six strains were deduced based on 16S rRNA sequences (Fig. 8A), the *pmo*A gene (Fig. 8B), and the NCBI database, which were affiliated with type I (*Methylococcus*) and type II (*Methylosinus* and *Methylocystis*) (Table 1). Similarities in *pmo*A gene sequences between isolated strains and amplicons (Fig. S8) indicated the reliability of the methanotroph-isolated strains identified in VMD rice fields. These methanotrophs were isolated between dilution levels of 10^–4^–10^–6^, which was similar to the series of cultivable-reported methanotrophs (10^–2^–10^–8^) (Takeda *et al.*, 2008; Pandit *et al.*, 2016; Rahalkar *et al.*, 2021). Moreover, the diluted level for isolating methanotrophs was consistent with the *pmo*A genes of soil samples collected in the present study (2.4×10^7^–2.6×10^8^ copies [g dry soil]^–1^), indicating that the dilution matched the estimation of methanotroph abundance in samples.

Although the present study showed that *Methylomicrobium* was the most abundant in the methanotrophic community structure of VMD rice fields, we were unable to isolate it from these fields. In addition, several methanotrophic genera, such as *Methylobacter*, *Methylocaldum*, *Methylogaea*, *Methylomonas*, *Methyloparacoccus*, *Methylomagnum*, and* Methylosarcina*, have been identified in VMD rice fields, but were not successfully isolated in the present study, suggesting the need for further research. Previous findings reported that *Methylomicrobium* was prevalent in rice ecosystems (Mohanty *et al.*, 2006; Noll *et al.*, 2008). However, the successful isolation of *Methylomicrobium* in rice fields has recently been reported by Rahalkar *et al.* (2021). Moreover, these strains were not isolated in the present study and are affiliated with type-I methanotrophs, which prefer low CH_4_ and high O_2_ concentrations (Megonigal *et al.*, 2003; Kambara *et al.*, 2022), suggesting the need for the optimization of cultivable conditions (*i.e.*, CH_4_ or NO_3_^–^) in further research.

*Methylosinus sporium* and *Methylocystis parva* corrig. were isolated from rice rhizospheric soil (Takeda *et al.*, 2008; Rahalkar *et al.*, 2021). *Methylococcus* strains have recently been isolated from rice fields (Awala *et al.*, 2023), albeit the genus has been widely identified in the methanotrophic community structure in rice rhizospheric soils (Lee‍ ‍*et al.*, 2014; Rahalkar *et al.*, 2021). To the best of our‍ ‍knowledge, several methanotroph genera have been isolated from rice fields: *Methylosinus*, *Methylocystis*, *Methylomonas*, *Methylobacter*, *Methylocucumis*, *Methylogaea*, *Methylococcaceae*, *Methylomagnum*, *Methylocaldum*, *Methyloterricola*, *Methylomicrobium*, *Methylotetracoccus*, and *Methylococcus* (Dianou and Adachi, 1999; Takeda *et al.*, 2008; Ferrando and Tarlera, 2009; Geymonat *et al.*, 2011; Dianou *et al.*, 2012; Islam *et al.*, 2015; Khalifa *et al.*, 2015; Pandit *et al.*, 2016; Pandit *et al.*, 2018; Ghashghavi *et al.*, 2019; Pandit and Rahalkar, 2019; Khatri *et al.*, 2020; Rahalkar *et al.*, 2021; Awala *et al.*, 2023; Kaise *et al.*, 2023). The present study isolated *Methylococcus*, *Methylosinus*, and *Methylocystis* from the soil of rhizospheric rice fields, which significantly contributes to the unknown representative strains in Vietnam and VMD. These strains may be vital for the development of strategies to reduce GHG emissions from rice production systems using indigenous methanotrophs identified in recent studies (Davamani *et al.*, 2020; Rani *et al.*, 2021).

## Conclusions

The present study exami­ned the methanotrophic community structure of rhizospheric paddy soil fertilized with BDE and SF in VMD and isolated several methanotroph strains from these fields. The results obtained revealed that MOB and *pmo*A gene abundance were lower in the BDE field than in the SF field. In addition, the long-term application of BDE did not affect the methanotrophic community structure of rice fields. In the methanotrophic community, 11 genera were identified in paddy soil, of which *Methylomicrobium* (Type-I) and *Methylosinus* (Type-II) were the most abundant. Type-I methanotrophs were more abundant than type-II methanotrophs. Although the difference in soil characteristics between BDE and SF fields was negligible, TOC and NO_3_^–^ were two primary factors affecting community structures. In methanotrophic isolation, we isolated three genera (*Methylococcus*, *Methylocystis*, and *Methylosinus*) with six methanotrophic strains from rice rhizospheric soil in VMD for the first time. These strains were similar to the amplicon of *pmo*A gene sequences in VMD rice fields. Overall, this study showed that the methanotroph community structure did not change with the application of BDE for three years. The isolation of methanotrophic strains is significant for the development of strategies to further reduce GHG emissions from rice production systems based on indigenous methanotrophs in VMD.

## Citation

Thao, H. V., Tarao, M., Takada, H., Nishizawa, T., Nam, T. S., Cong, N. V., and Xuan, D. T. (2024) Methanotrophic Communities and Cultivation of Methanotrophs from Rice Paddy Fields Fertilized with Pig-livestock Biogas Digestive Effluent and Synthetic Fertilizer in the Vietnamese Mekong Delta. *Microbes Environ ***39**: ME24021.

https://doi.org/10.1264/jsme2.ME24021

## Supplementary Material

Supplementary Material

## Figures and Tables

**Table 1.** Methanotroph strains isolated from paddy soils fertilized with BDE and SF

Fields	Genera	Accession number	16S rRNA gene sequence, identity	Partial *pmo*A gene sequence, identity
BE1 (10^–4^)	*Methylococcus* sp.	PP178148^†^ PP182324^‡^	*Methylococcus capsulatus* Texas=ATCC 19069^T^ (NR042183), 98.47%	*Methylococcus thermophilus* AQ4^T^ (OR004532), 100%
BE2 (10^–5^)	*Methylocystis* sp.	PP218659^†^ PP239038^‡^	*Methylocystis parvus* OBBP^T^ (NR044946), 97.78%	*Methylocystis* sp. IMET 10484^T^ (AJ458998), 98.50%
SF1 (10^–4^)	*Methylosinus* sp.	PP218660^†^ PP239039^‡^	*Methylosinus sporium* NCIMB 11126^T^ (NR026512), 98.93%	*Methylosinus sporium* KS17^T^ (AJ459031), 98.74%
SF2 (10^–5^)	*Methylosinus* sp.	PP218670^†^ PP239041^‡^	*Methylosinus sporium* NCIMB 11126^T^ (NR026512), 99.01%	*Methylosinus* sp. B4S^T^ (AB683146), 99.57%
SF3 (10^–6^)	*Methylococcus* sp.	PP218671^†^ PP239042^‡^	*Methylococcus capsulatus* Texas^T^=ATCC 19069 (NR042183), 98.51%	*Methylococcus thermophilus* AQ4^T^ (OR004532), 100%
SF4 (10^–5^)	*Methylosinus* sp.	PP218672^†^ PP239040^‡^	*Methylosinus sporium* NCIMB 11126^T^ (NR026512), 98.99%	*Methylosinus sporium* B4S^T^ (AB683146), 99.58%

The numbers in parentheses indicate the dilution of soil from which the isolates were obtained. ^†^ and ^‡^ indicate the accession numbers of the 16S rRNA and *pmo*A gene sequences submitted to NCBI GenBank, respectively.
