# Supplementary material for: Methanotrophic Communities and Cultivation of Methanotrophs from Rice Paddy Fields Fertilized with Pig-livestock Biogas Digestive Effluent and Synthetic Fertilizer in the Vietnamese Mekong Delta
Source: Microbes Environ. 2024 Oct 2;39(4):ME24021. doi: 10.1264/jsme2.ME24021 (PMC11821765; doi:10.1264/jsme2.ME24021)
Supplement: Supplementary file 1 — Supplementary Material [file 39_24021_s1.pdf]

Supplementary Material

Methanotrophic Communities and Cultivation of Methanotrophs from Rice Paddy Fields Fertilized with Pig-livestock Biogas Digestive Effluent and Synthetic Fertilizer in the Vietnamese Mekong Delta

Huynh Van Thao[1,2], Mitsunori Tarao[1*], Hideshige Takada[1], Tomoyasu Nishizawa[3], Tran Sy Nam[2], Nguyen Van Cong[2], Do Thi Xuan[4]

[1] United Graduate School of Agricultural Science, Tokyo University of Agriculture and Technology, Tokyo 183-8506, Japan

[2] Department of Environmental Sciences, College of Environment and Natural Resources, Can Tho University, 3/2 street, Can Tho city 900000, Viet Nam

[3] Ibaraki University College of Agriculture, 3-21-1 Chuou, Ami-machi, Ibaraki, Japan

[4] Department of Microbial Technology, Institute of Food and Biotechnology, Can Tho University, 3/2 street, Can Tho city 900000, Viet Nam

*** Correspondence:**

Mitsunori Tarao

tarao@cc.tuat.ac.jp

Supplementary Tables

Table S1. Chemical properties of diluted biogas effluent irrigated for rice paddies fields.

Properties	Dry	Wet
pH (H_2O)	7.37 ± 0.17	7.40 ± 0.20
EC (mS cm^{-1})	26±0.42	22±0.27
TOC (mg C L^{-1})	283±34.6	266±29.2
TN (mg N L^{-1})	278±34.2	234±37.5
NH_4^+ (mg N L^{-1})	203 ± 39.1	196± 33.6
NO_3^- + NO_2^- (mg N L^{-1})	0.18 ± 0.05	0.31 ± 0.04
Total P (mg P L^{-1})	115 ± 6.31	110 ± 10.80
Total K (mg K L^{-1})	285±28.3	215±16.9

Data are presented by mean ± standard deviation (*n* = 3). Biogas effluent was applied in two seasons, comprising dry and wet seasons.

Table S2. Soil physiochemical properties of paddy fields applied with biogas digestive effluent (BDE) or synthetic fertilizer (SF).

Treatments	pH	EC ($\mu S\ cm^{-1}$)	TOC ($g\ C\ kg^{-1}$)	TC ($g\ C\ kg^{-1}$)	TN ($g\ N\ kg^{-1}$)	NH_4^+ ($mg\ N\ kg^{-1}$)	NO_3^- ($mg\ N\ kg^{-1}$)	PNM ($mg\ N\ kg^{-1}$)
Seasons								
Dry	5.24±0.29	297±99	37.0±3.26	42.2±2.34	3.08±0.18	19.6±4.04	2.77±1.07	63.9±11.8
Wet	5.26±0.26	296±113	36.9±3.74	42.3±3.92	3.09±0.23	18.7±5.4	3.05±1.21	62.3±19.1
Paddy fields								
BDE	5.19±0.17	379±81.4	36.9±4.32	42.4±3.4	3.00±0.19	17.0±3.9	2.82±1.24	71.4±16.3
SF	5.31±0.34	214±30.8	37.0±2.44	42.0±3.0	3.17±0.20	21.3±4.25	3.00±1.05	54.7±9.5

Data are presented by mean ± standard deviation ($n = 3$).

Table S3. Summary of permutation test for all RDAs

	DF	Variance	***F***	***P***
RDA1	1	0.07	9.81	**
RDA2	1	0.02	2.75	0.28
RDA3	1	0.01	1.43	0.69
RDA4	1	0.00	0.26	1.00
RDA5	1	0.00	0.10	1.00
Residual	11	0.11		

Significant ANOVA differences: ***$P < 0.001$, **$P < 0.01$, *$P < 0.05$, †$P < 0.1$

Table S4. Summary of permutation test for environmental variables on methanotrophic genus levels

	**DF**	**Variance**	***F***	***P***
EC	1	0.02	3.11	*
TOC	1	0.03	3.94	**
TC	1	0.01	0.71	0.58
PMN	1	0.01	1.83	0.13
NO_3^-	1	0.04	4.76	**
Residual	14	0.11		

Significant differences: ***$P < 0.001$, **$P < 0.01$, *$P < 0.05$, †P < 0.1

Supplementary Figures

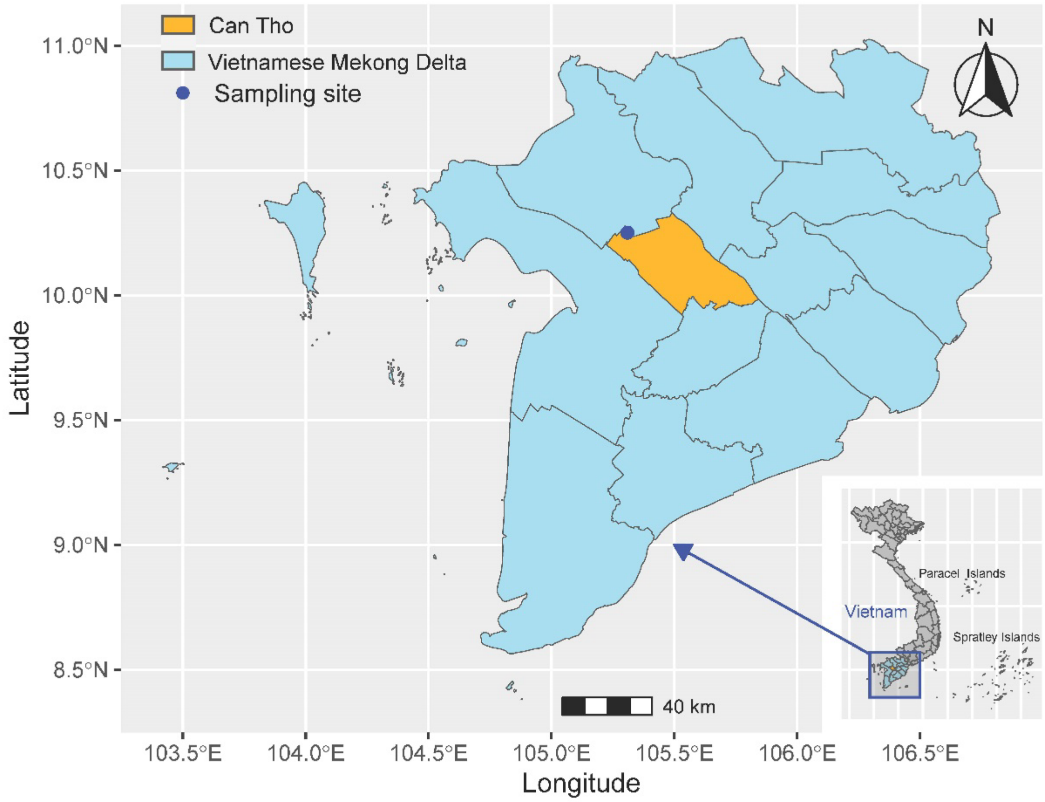

Fig. S1. Map of study site in Vinh Thanh district, Can Tho city, Vietnamese Mekong Delta.

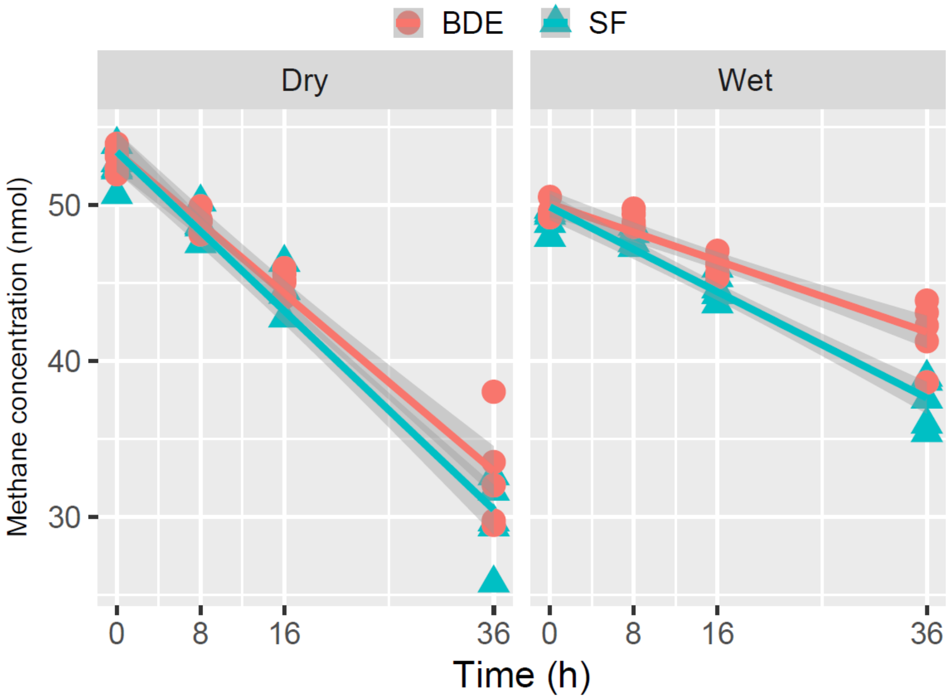

Fig. S2. Methane concentration change overtime in soil slurries

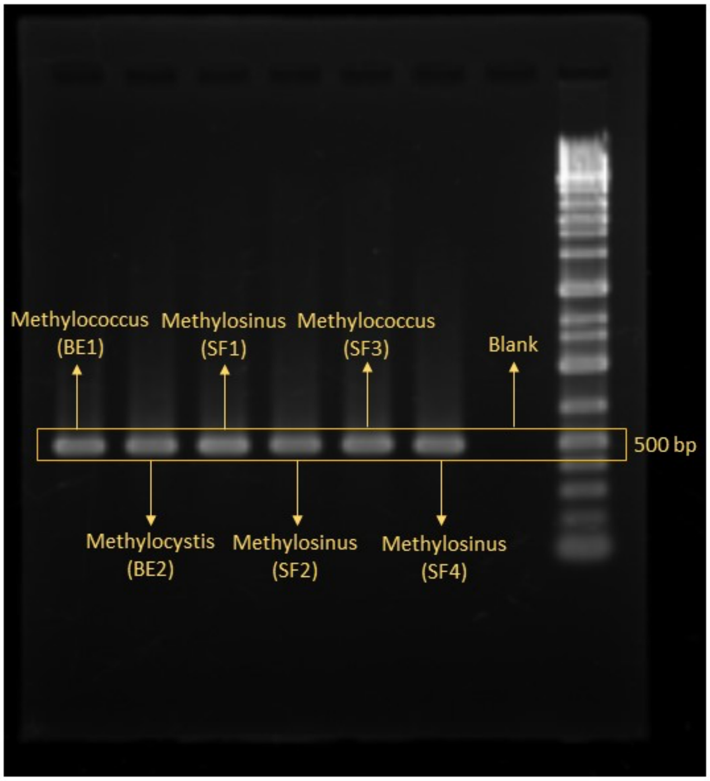

Fig. S3. Gel electrophoresis of isolated methanotrophs amplified with *pmo*A gene (A189f and mb661).

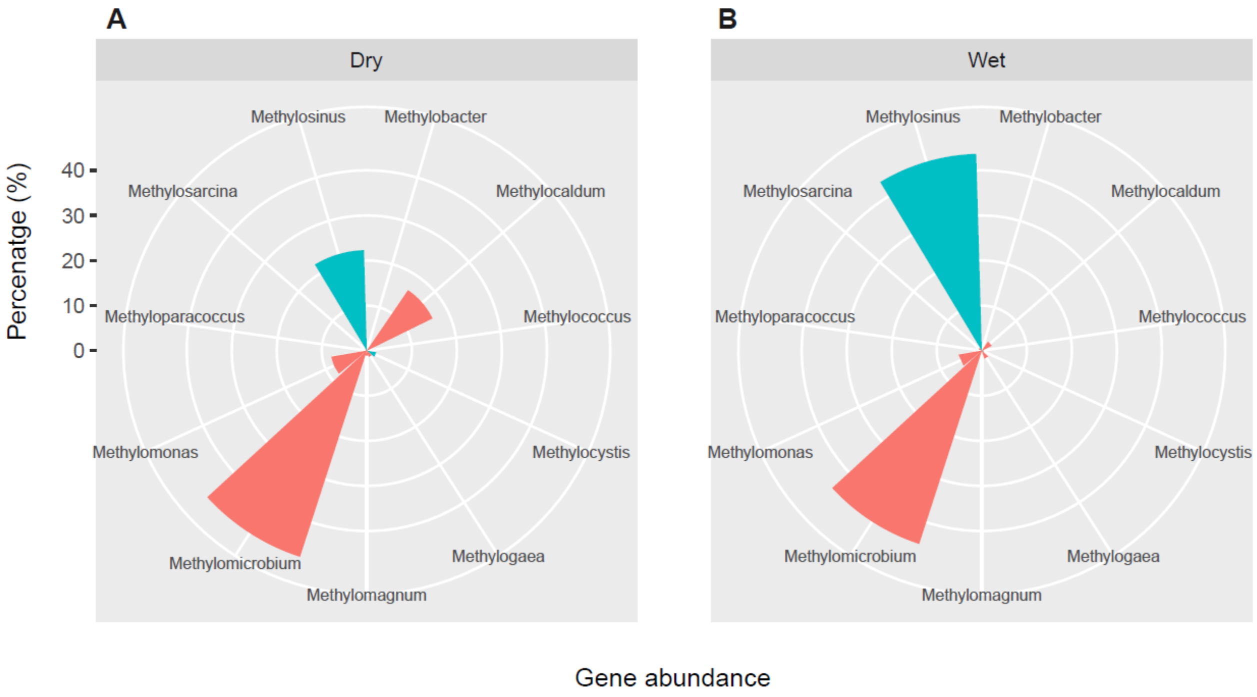

Fig. S4. The percentage of gene abundance in methanotrophic community under differences in wet and dry seasons

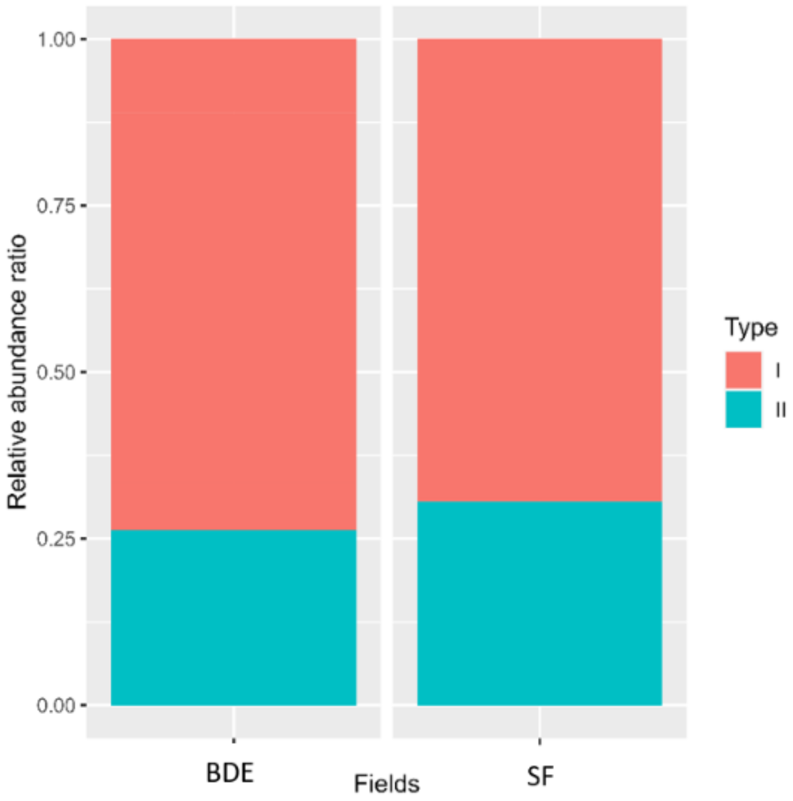

Fig. S5. Percentage of methanotrophic types at genus levels.

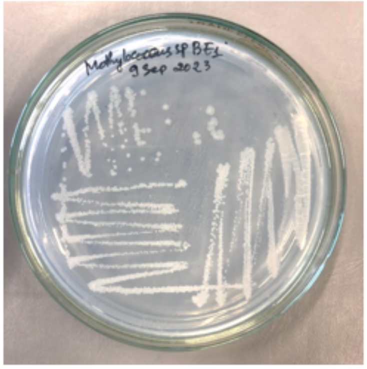

Methylococus sp. strain BE1

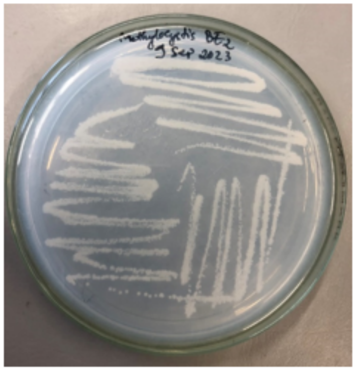

Methylocystis sp. strain BE2

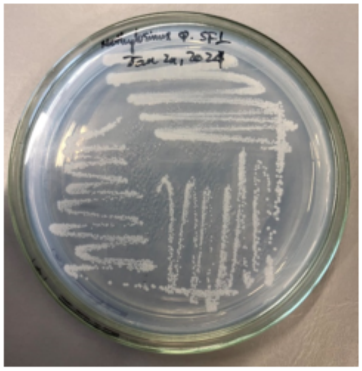

Methylosinus sp. strain SF1

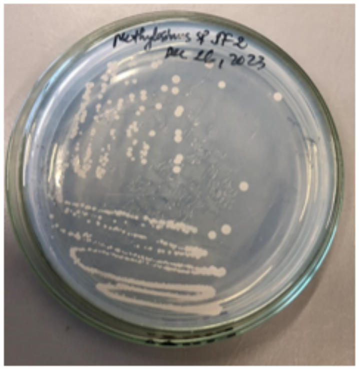

Methylosinus sp. strain SF2

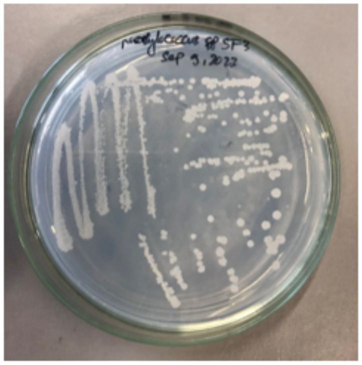

Methylococus sp. strain SF3

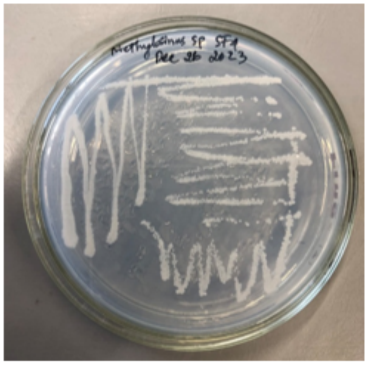

Methylosinus sp. strain SF4

Fig. S6. Growth of isolated methanotroph on solid media.

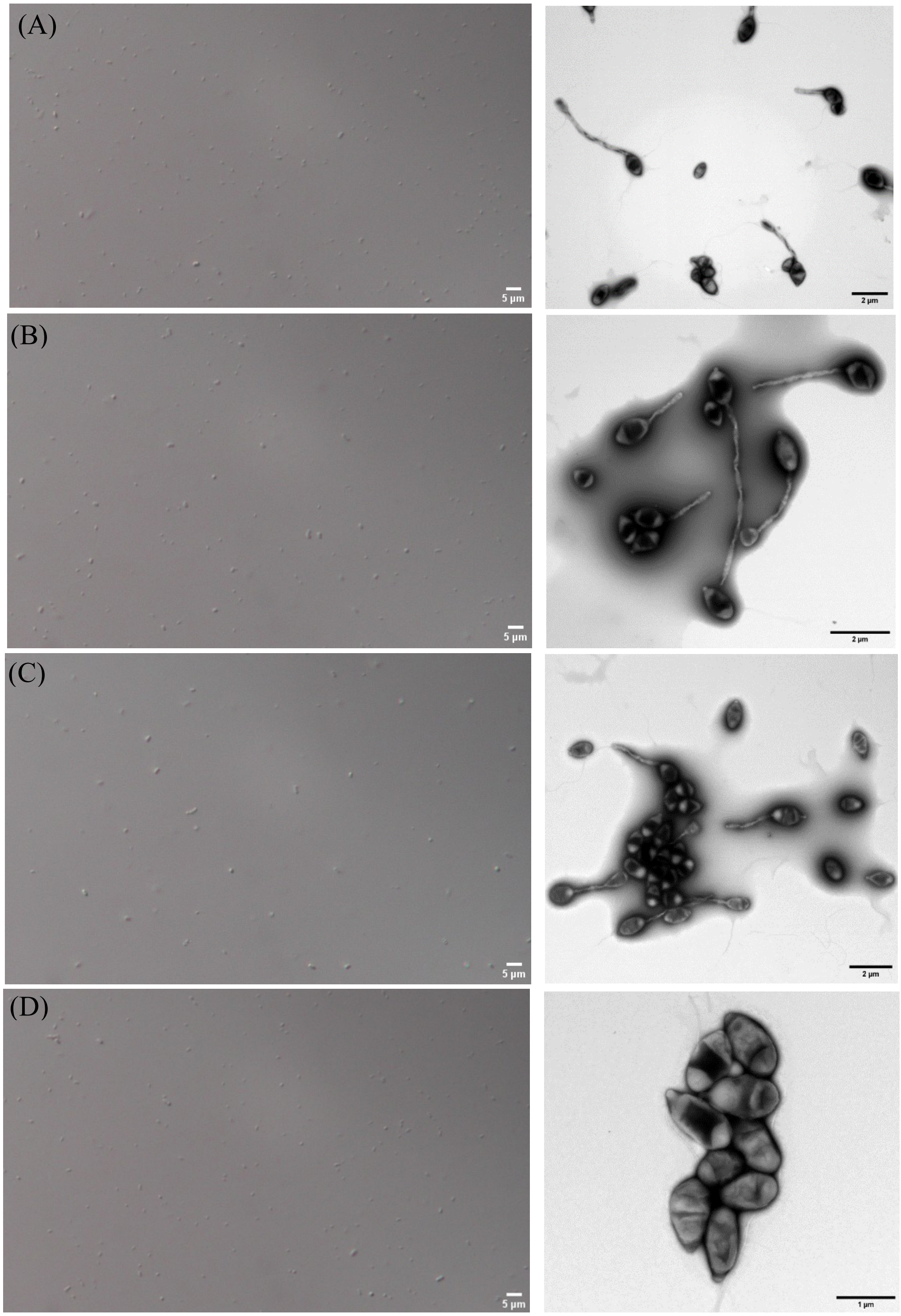

Fig. S7. Differential interference contrast (DIC) microscope images and transmission electron microscopic (TEM) images of isolated methanotrophs observed under JEM-1400Flash electron microscopes (JEOL. CO., Ltd) (right). (A), (B), (C), and (D) show strains of *Methylosinus* sp. SF1, *Methylosinus* sp. SF2, *Methylosinus* sp. SF4, and *Methylocystis* sp. BE2, respectively. No microscopic images of *Methylococcus* sp. strains are shown as the strains were not recovered from the glycerol stocks kept at -80 ºC.

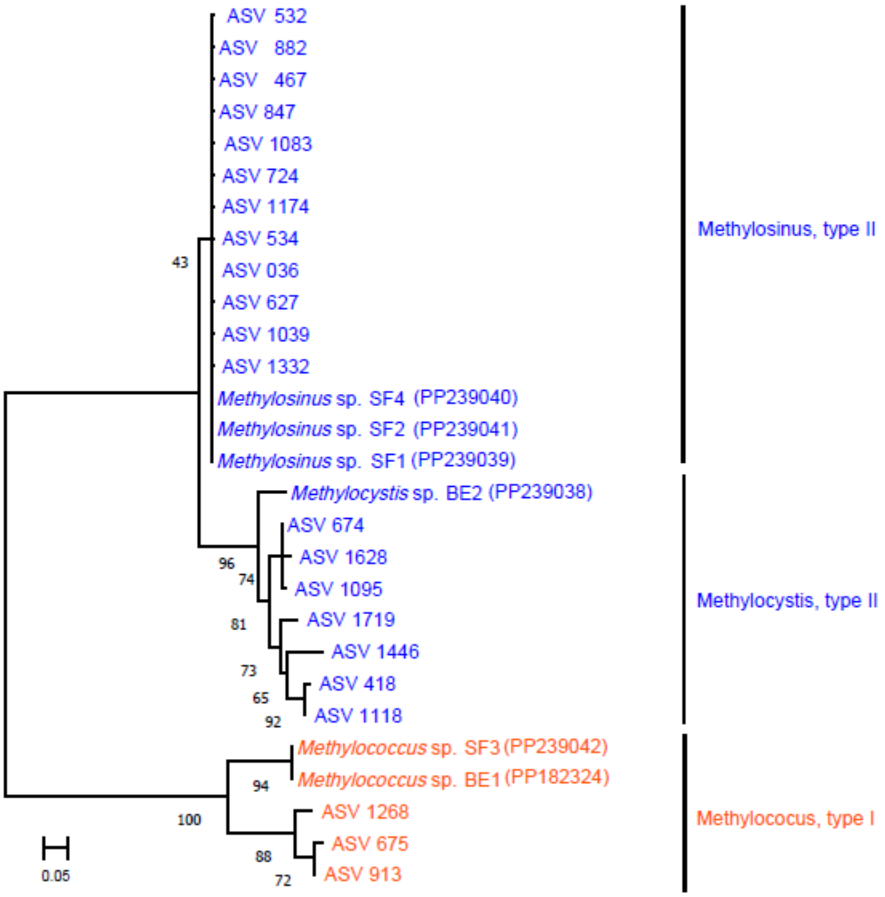

Fig. S8. Neighbor-Joining trees showing the polygenetic tree of the *pmo*A gene sequences of methanotroph strains isolated from the VMD's rice paddy fields with their closest member identified in the amplicon of *pmo*A gene sequences.

Isolated strains are highlighted with orange (type-I methanotrophs) and blue (type-II methanotrophs) colors. All ambiguous positions were removed for each sequence pair. Bootstrap (1000 interactions) values are shown. The scale bar indicates 5% estimated sequence divergence.
